# Humoral immune reconstitution following therapy with daratumumab, carfilzomib, lenalidomide, and dexamethasone (Dara‐KRd), autologous hematopoietic cell transplantation, and measurable residual disease‐response‐adapted treatment cessation

**DOI:** 10.1002/jha2.736

**Published:** 2023-06-22

**Authors:** Rebecca W. Silbermann, Timothy Martin Schmidt, Susan Bal, Binod Dhakal, Bhagirathbhai Dholaria, Eden Biltibo, Saurabh Chhabra, Smith Giri, Kelly N. Godby, Sonia Gowda, Eva Medvedova, Robert F. Cornell, Natalie S. Callander, Luciano J. Costa

**Affiliations:** ^1^ Knight Cancer Institute Oregon Health and Science University Portland Oregon USA; ^2^ Carbone Cancer Center University of Wisconsin Madison Wisconsin USA; ^3^ University of Alabama at Birmingham Birmingham AL UK; ^4^ Division of Hematology/Oncology, Department of Medicine Medical College of Wisconsin Milwaukee Wisconsin USA; ^5^ Vanderbilt University Medical Center Nashville TN USA; ^6^ Institute for Cancer Outcomes and Survivorship University of Alabama at Birmingham Birmingham AL UK; ^7^ Department of Internal Medicine Oregon Health and Science University Portland Oregon USA

**Keywords:** immunodeficiency, measurable residual disease, multiple myeloma

## Abstract

Quadruplet induction, autologous hematopoietic cell transplant (AHCT), and measurable residual disease (MRD) response‐adapted consolidation yield an unprecedented depth of response in newly diagnosed multiple myeloma. Patients treated on MASTER (NCT03224507) ceased therapy and entered active surveillance (MRD‐SURE) after achieving MRD negativity. This study characterizes quantitative changes in the immunoglobulin (Ig) gene repertoire by next‐generation sequencing and serum gamma globulin levels. Quadruplet therapy leads to profound hypogammaglobulinemia and reduction in the Ig gene repertoire. Immune reconstitution (IR) is delayed in patients who received post‐AHCT consolidation compared to those who do not. Eighteen months after treatment cessation, there was no statistically significant difference between the groups.

1

Multiple myeloma (MM), the second most common hematologic malignancy, is routinely managed with continuous therapy and is considered an incurable disease. Rapid drug development has led to a dramatic improvement in the overall survival of most MM patients over the past two decades. Modern regimens combining immunomodulatory (IMiD) drugs, proteasome inhibitors (PI), and monoclonal antibodies (mAb) are generally well tolerated and allow patients to achieve deep and durable responses associated with improved survival, particularly with the achievement of undetectable measurable residual disease (MRD) [1]. Quadruplet induction therapies, including a CD38 mAb, IMiD, PI, and steroid followed by autologous hematopoietic cell transplant (AHCT), yield an unprecedented depth of response and MRD‐negativity among patients with newly diagnosed MM [2, 3, 4], and patients who achieve sustained MRD‐negativity after initial therapy have a very low rate of disease progression [5, 6]. While the positive impact of quadruplet therapy on the depth of disease response is clear, the effect of intensive therapy on immune function and immunoparesis recovery, a proposed positive predictor of long‐term progression and survival [7, 8], has not been described.

The MASTER trial (NCT03224507) evaluated the feasibility of intensive therapy followed by MRD‐adapted consolidation and/or treatment cessation with MRD surveillance. This allowed some patients to enjoy a lengthy treatment‐free interval and provides a unique opportunity to evaluate immune reconstitution in patients with a deep disease response who are observed off of therapy. The study procedures and results of the primary analysis have been previously reported in detail [3]. Briefly, patients received induction with daratumumab, carfilzomib, lenalidomide, and dexamethasone (DaraKRd; 4 cycles), followed by AHCT and 0, 4, or 8 cycles of Dara‐KRd consolidation guided by serial assessments of bone marrow MRD by next‐generation sequencing (NGS, ClonoSEQ®). Patients with two consecutive negative MRD assessments at a depth of < 10^−5^ ceased therapy and entered active surveillance for MRD resurgence (MRD‐SURE).

In this study, we retrospectively analyzed the impact of DaraKRd and AHCT on biomarkers of humoral immunity among patients in MASTER for whom complete data were available at key time points. The primary objective of this study was to characterize quantitative changes in the repertoire of immunoglobulin (Ig) genes (IgH, IgK, and IgL) as assessed by NGS at each MRD assessment throughout treatment and post‐treatment surveillance. For all patients, MRD status was assessed after 4 cycles of induction therapy, after AHCT, and after each 4‐cycle block of Dara‐KRd consolidation. Among patients entering MRD‐SURE, evaluation for MRD resurgence was assessed 24 and 72 weeks after discontinuation of therapy. A schema of the MASTER trial, color‐coded for the different time periods and MRD analyses, is shown in Figure 1A.

**FIGURE 1:**
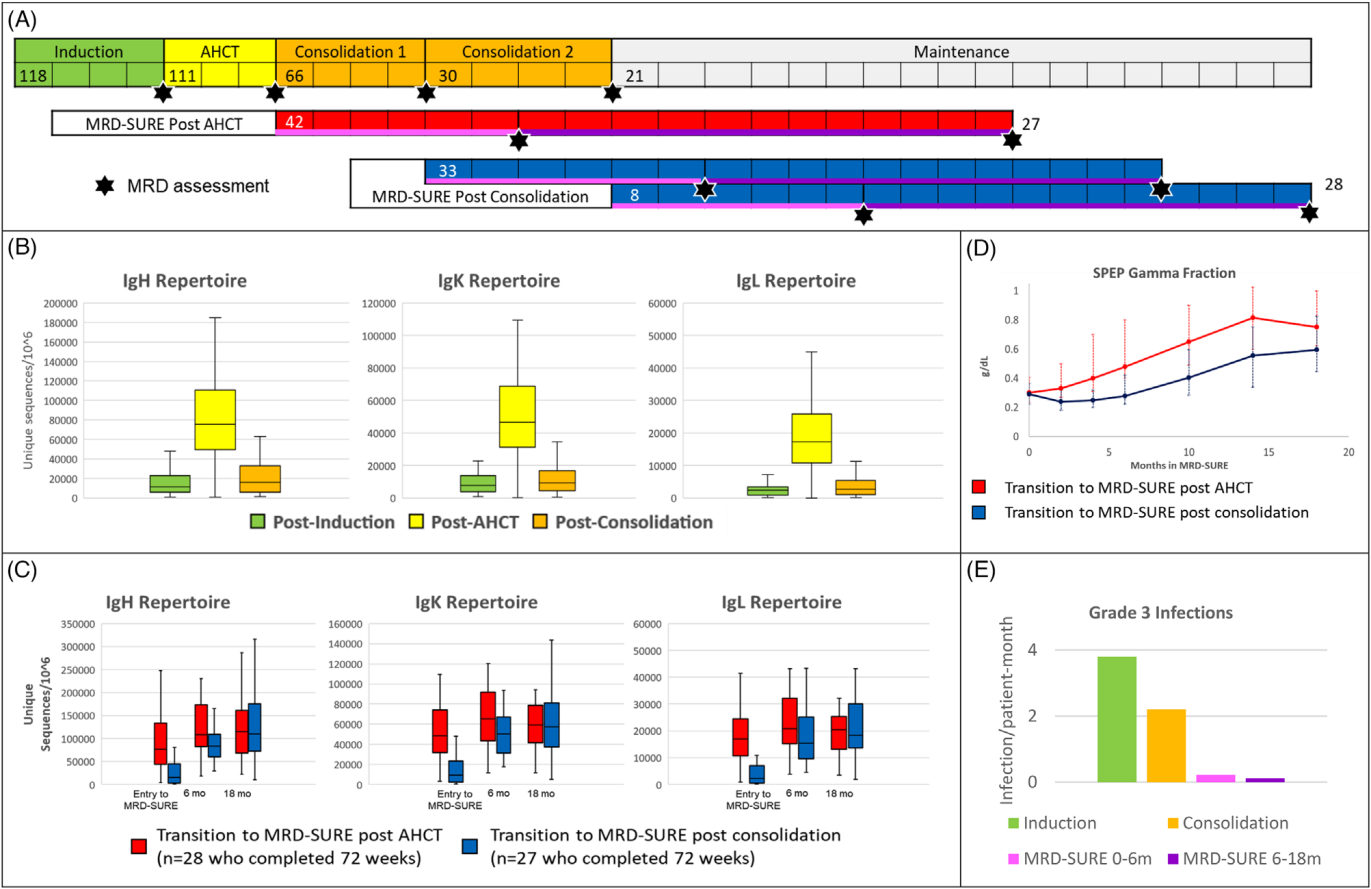
(A) Color‐coded MASTER schema. The number of patients in MASTER who remained on treatment with evaluable MRD results are listed at each phase of treatment and/or surveillance. Phases and MRD assessments are represented in different colors, which are then used in panels B–E. Each block is 1 month. (B) Unique immunoglobulin sequences as measured by NGS while on treatment. Data are shown for *n* = 63 patients who received post‐AHCT consolidation and had NGS data available at the end of induction, AHCT, and consolidation, regardless of MRD status and disposition. (C) Unique immunoglobulin sequences among patients entering MRD‐SURE immediately after AHCT (red) and after post‐AHCT consolidation (blue), for whom evaluable data sets were available. (D) Serum gamma globulin levels among patients entering MRD‐SURE immediately after AHCT (red) and after post‐AHCT consolidation (blue). (E) Grade 3+ infections during treatment with DaraKRd and MRD‐SURE. Infections occurring within the first 100 days post‐AHCT were excluded due to confounding by the immune suppression related to AHCT and patients on maintenance were excluded due to lack of data. Abbreviations: AHCT, autologous hematopoietic cell transplant; MRD, measurable residual disease; NGS, next‐generation sequencing.

We also individually reviewed charts for evidence of grade ≥3 infections and evaluated routinely measured parameters that are associated with immune reconstitution. Due to differences in the standard laboratory assessments of patients not on therapy, this analysis was limited to laboratory parameters specified by the protocol and consisted of serum gamma globulin levels (as measured on serum protein electrophoresis [SPEP]) and leukocyte subset populations (as assessed by routine complete blood count) at key time points.

Bone marrow aspirate samples were evaluated by NGS by Adaptive Biotechnologies. Unique IgH, IgK, or IgL sequences in each patient sample were identified and quantified per standard ClonoSeq® methodology [9], and these data were extracted from the report appendix. Data are expressed as median unique sequences (mus)/10^6^.

We first evaluated the impact of Dara‐KRd on humoral immunity in the peri‐transplant time period. Sixty‐three patients who underwent induction, AHCT, and at least 4 cycles of Dara‐KRd consolidation had evaluable data sets. We found that the Ig gene repertoire expands following AHCT and contracts following post‐AHCT consolidation, as shown in Figure 1B. After induction, the number of median unique sequences (mus)/10^6^ for IgH, IgK, and IgL were 11,330, 7657, and 2314, respectively. Ninety days after transplant, IgH, IgK, and IgL mus/10^6^ expanded to 75,375, 46,541, and 17,207, respectively. Patients receiving post‐transplant consolidation had a reduced Ig diversity with IgH, IgK, and IgL mus/10^6^ of 15,795, 9246, and 2750, respectively.

Immune reconstitution patterns were then evaluated among patients who entered MRD‐SURE and continued without disease progression after 72 weeks of treatment‐free observation. Data were not collected for patients who did not enter MRD‐SURE. Complete data sets were available for 28 patients who entered MRD‐SURE immediately after AHCT and 27 patients who entered MRD‐SURE after additional consolidation. Ig diversity as measured by the number of unique sequences by NGS was significantly higher for patients entering MRD‐SURE after AHCT compared to those entering MRD‐SURE after Dara‐KRd consolidation (IgH 76,266 v 15,082; IgK 48,479 v 9246; IgL 16,980 v 2292; *p* < 0.001 for all). After 6 months in MRD‐SURE, the Ig repertoire expanded in both groups, although mus remained higher in the post‐AHCT group (IgH 107,715 v 83,954, *p* = 0.037; IgK 65,099 v 50,167, *p* = 0.050; IgL 20,771 v 15,406, *p* = 0.010). After 18 months of MRD‐SURE, there was no significant difference in immune repertoire as measured by NGS between groups. These data are demonstrated in Figure 1C.

Patients entering MRD‐SURE after AHCT had significantly higher gamma globulin levels as measured by SPEP at each measured time point compared to those receiving Dara‐KRd consolidation. Notably, a rise in the mean gamma fraction level was seen immediately for patients entering MRD‐SURE after AHCT, whereas there was a 6‐month delay in Ig recovery for patients who received post‐AHCT consolidation (Figure 1D). These data, taken together with the Ig sequence repertoire, suggest that Dara‐KRd consolidation delays immune reconstitution and that the immune repertoire expands prior to gamma globulin recovery. We also evaluated trends in leukocyte subsets, including neutrophils, lymphocytes, and monocytes. No significant trends were noted in these analyses (data not shown).

Finally, we extracted clinically significant infections from the clinical trial database, supplementing the available data with a chart review. We focused on grade ≥3 infections, due to the low capture rate of grade 1–2 infections for patients who were off therapy and, therefore, monitored less frequently. Infections in the first 100 days after AHCT were omitted from this analysis. The frequency of grade≥3 infections while on Dara‐KRd induction was 3.8/100*patient*month and 2.2/100*patient*month during post‐AHCT consolidation (overall 2.9/100*patient*month while on Dara‐KRd). The risk of grade≥3 infection reduced substantially to 0.22/100*patient*month during the first 6 months and 0.12/100*patient*month during subsequent 12 months of MRD‐SURE (overall 0.15/100*patient*month while off therapy, *p* = 0.0001 vs. risk while on Dara‐KRd). A graphical representation is shown in Figure 1E.

To our knowledge, these data are the first description of patterns of humoral immune reconstitution among patients with MM who discontinue therapy after achieving sustained MRD‐negativity. Our use of NGS to describe the recovery of the immune microenvironment following intensive therapy is also novel. We have shown that quadruplet, anti‐CD38 mAb‐containing therapy leads to profound hypogammaglobulinemia and reduction in the Ig gene repertoire, which promptly recovers after AHCT. Patients who received post‐AHCT consolidation had delayed immune reconstitution compared to those who did not; however, the Ig repertoire steadily recovered with no statistically significant difference by 18 months after treatment cessation. Fortunately, grade ≥3 infection risk was very low after treatment cessation, potentially underscored by our observation that the Ig gene repertoire expands prior to recovery of gamma globulin levels. Protocols for antimicrobial prophylaxis, post‐transplant vaccination, and Ig replacement for patients with hypogammaglobulinemia were not standardized in MASTER, and very few patients received supplemental gamma globulin infusions. Patients who received Ig supplementation were censored for both infection and immune reconstitution outcomes at the time of administration [10].

Our study has several limitations, including a small sample size, incomplete assessment of total Ig levels and immune subsets, and relatively short follow‐up of patients on MRD‐SURE. We are unable to compare immune reconstitution by NGS to patients on lenalidomide maintenance as patients who did not enter MRD‐SURE did not have ongoing MRD surveillance testing. Furthermore, we are unable to comment on if the Ig diversity observed is comparable to nonmyeloma populations or patient populations receiving standard courses of quadruplet induction therapy followed by AHCT, as there is no established reference range for “normal” Ig sequences by NGS. However, our data introduce a novel application for NGS assessment of the myeloma immune microenvironment beyond MRD that may be included in the design of future studies.

## AUTHOR CONTRIBUTIONS

RWS, TMS, LJC, BD (MCW), and SB designed the study and performed the data analysis. RWS and TMS prepared the manuscript. All authors participated in data collection and manuscript approval.

## FUNDING INFORMATION

N/A, Amgen, and Janssen provided material support for the MASTER study but were not directly involved in study design, conduct, data collection, data analysis, or manuscript preparation.

## CONFLICT OF INTEREST STATEMENT

RWS served as a consultant for Janssen and Sanofi. TMS served as a consultant for Janssen and Sanofi. SB served as a consultant for Adaptive Biotechnologies. BD (MCW) has received honoraria from Bristol Myers Squibb and Karyopharm, served as a consultant for Amgen, GlaxoSmithKline, Janssen, Natera, and Sanofi, and received research funding from GlaxoSmithKline and Sanofi. BD (VU) received research funding from Angiocrine, Poseida, Takeda, Pfizer, Janssen, MEI, Medtemplate, Molecular Templates, and Wugen, served as a consultant for BEAM Therapeutics, Gamida Cell, Jazz, and Arivan, and received honoraria from MJH Biosciences, Taylor and Francis, and Wiley. SC reports research funding from Janssen, Sanofi, and Amgen and received honoraria from GlaxoSmithKline. RFC is currently employed by Abbvie. SG (UAB) received honoraria from CareVive and OncLive and research funding from CareVive and Pack Health. LJC reports consultancy for Janssen, Amgen, Sanofi, BMS, and Adaptive Biotechnologies, honoraria from Janssen, Amgen, Sanofi, BMS, Adaptive Biotechnologies, and Abbvie, and research funding from Janssen, Amgen, BMS, and Abbvie. EB, KG, SG (OHSU), EM, and NSC report no competing interests.

2

## ETHICS APPROVAL STATEMENT

The study was conducted in accordance with the International Council for Harmonization Good Clinical Practice Guidelines and the principles of the 2013 Declaration of Helsinki. Institutional review boards approved the Protocol (online only) and appropriate related documents.

## PATIENT CONSENT STATEMENT

All patients provided written informed consent for participation.

## CLINICAL TRIAL REGISTRATION (INCLUDING TRIAL NUMBER)

The study is registered on ClinicalTrials.gov (NCT03224507).

## Data Availability

Data available on request from the authors.
